# Supplementary material for: Deciphering the Polyglucosan Accumulation Present in Lafora Disease Using an Astrocytic Cellular Model
Source: Int J Mol Sci. 2023 Mar 23;24(7):6020. doi: 10.3390/ijms24076020 (PMC10094345; doi:10.3390/ijms24076020)
Supplement: Supplementary file 1 [file ijms-24-06020-s001.zip › ijms-2274377-supplementary.pdf]

Supplementary Table S1

Full list of the differential changes in the levels of intermediate metabolites in control and *Epm2b-/-* astrocytes growing in DMEM + 10% FBS medium with no extra glucose added for 24 h. The fold change and the p-values are indicated (n=4). Values are arranged according to FC. *Indicates a compound that has not been confirmed based on a standard, but we are confident in its identity. **Indicates a compound for which a standard is not available, but we are reasonably confident in its identity or the information provided.

**Metabolite**	**FC *Epm2b-/-*/ Control**	**p value *Epm2b-/-*/ Control**
fructose 1,6-diphosphate/glucose 1,6-diphosphate/myo-inositol diphosphates	5,19	0,023
glucose 6-phosphate	3,77	0,023
glucosamine-6-phosphate	3,22	0,417
fructose-6-phosphate	2,56	0,596
dihydroxyacetone phosphate (DHAP)	2,44	0,192
aconitate [cis or trans]	2,40	0,195
dihomo-linoleoylcarnitine (C20:2)*	2,28	0,185
gluconate	2,27	0,063
N-carbamoylaspartate	2,25	0,323
6-phosphogluconate	2,18	0,336
1-ribosyl-imidazoleacetate*	2,08	0,027
citrate	1,90	0,243
adenosine-5'-diphosphoglucose	1,88	0,285
glucose	1,87	0,247
nisinate (24:6n3)	1,70	0,652
1-arachidonoyl-GPC (20:4n6)*	1,68	0,544
2'-deoxyguanosine	1,66	0,079
2-stearoyl-GPE (18:0)*	1,65	0,682
orotate	1,62	0,478
N-stearoyltaurine	1,62	0,554
cerotoylcarnitine (C26)*	1,56	0,265
N-oleoyltaurine	1,55	0,657
sedoheptulose-7-phosphate	1,54	0,113
mannose	1,54	0,247
isovalerylcarnitine (C5)	1,54	0,519
erucate (22:1n9)	1,54	0,571
1-arachidonoyl-GPE (20:4n6)*	1,53	0,554
2'-deoxyinosine	1,52	0,170
glutathione, oxidized (GSSG)	1,48	0,261
acetylphosphate	1,48	0,422

inositol 1-phosphate (I1P)	1,48	0,622
adenosine 5'-diphosphate (ADP)	1,47	0,700
4-methyl-2-oxopentanoate	1,46	0,344
eicosapentaenoate (EPA; 20:5n3)	1,46	0,654
phosphoenolpyruvate (PEP)	1,43	0,455
1-stearoyl-2-docosahexaenoyl-GPE (18:0/22:6)*	1,42	0,212
2-hydroxypalmitate	1,42	0,364
myristate (14:0)	1,42	0,409
2-methylbutyrylcarnitine (C5)	1,42	0,586
palmitoyl ethanolamide	1,42	0,772
dihomo-linoleate (20:2n6)	1,40	0,723
N-acetylglucosamine/N-acetylgalactosamine	1,39	0,356
linoleoyl ethanolamide	1,39	0,982
1-oleoyl-GPS (18:1)	1,37	0,687
heneicosapentaenoate (21:5n3)	1,37	0,767
docosatrienoate (22:3n6)*	1,37	0,830
docosadienoate (22:2n6)	1,37	0,935
2-palmitoyl-GPC (16:0)*	1,36	0,782
aspartate	1,35	0,193
docosahexaenoate (DHA; 22:6n3)	1,35	0,652
glucuronate	1,34	0,134
2-hydroxystearate	1,34	0,428
1-stearoyl-GPS (18:0)*	1,34	0,656
1-oleoyl-GPG (18:1)*	1,34	0,823
3-formylindole	1,33	0,256
galactose 1-phosphate	1,33	0,329
docosapentaenoate (n3 DPA; 22:5n3)	1,33	0,791
eicosenoate (20:1)	1,33	0,801
1-palmitoyl-GPS (16:0)*	1,32	0,617
palmitoleate (16:1n7)	1,32	0,705
arachidonate (20:4n6)	1,32	0,732
nicotinamide riboside	1,31	0,155
2-oxoarginine*	1,31	0,210
sedoheptulose	1,31	0,242
oleoyl ethanolamide	1,31	0,928
heptadecasphingosine (d17:1)	1,30	0,266
3-phosphoglycerate	1,30	0,569
stearate (18:0)	1,29	0,541
5-hydroxyindoleacetate	1,29	0,755
mead acid (20:3n9)	1,29	0,770
1-palmitoyl-GPG (16:0)*	1,29	0,780

1-stearoyl-GPI (18:0)	1,29	0,897
glycerate	1,28	0,672
1-palmitoylglycerol (16:0)	1,28	0,683
pentadecanoate (15:0)	1,27	0,567
margarate (17:0)	1,27	0,668
10-nonadecenoate (19:1n9)	1,27	0,810
3-sialyllactose	1,27	0,828
nonadecanoate (19:0)	1,27	0,838
valylglycine	1,26	0,249
N-acetylglucosaminylasparagine	1,26	0,338
adrenate (22:4n6)	1,26	0,888
taurolithocholate	1,25	0,628
ribose	1,24	0,182
glycosyl-N-palmitoyl-sphingosine (d18:1/16:0)	1,24	0,202
1-palmitoleoyl-2-oleoyl-GPC (16:1/18:1)*	1,24	0,326
1,2-dioleoyl-GPI (18:1/18:1)	1,24	0,358
spermine	1,24	0,425
isoleucylglycine	1,23	0,279
3-hydroxyadipate*	1,23	0,363
guanosine 5'-diphospho-fucose	1,23	0,404
(S)-3-hydroxybutyrylcarnitine	1,23	0,411
glycylleucine	1,22	0,361
pterin	1,22	0,384
succinylcarnitine (C4-DC)	1,22	0,446
10-heptadecenoate (17:1n7)	1,22	0,778
N-monomethylarginine	1,21	0,215
fumarate	1,21	0,331
fructosyllysine	1,21	0,421
2-palmitoylglycerol (16:0)	1,21	0,784
glutathione, reduced (GSH)	1,20	0,261
1-(1-enyl-palmitoyl)-2-linoleoyl-GPE (P-16:0/18:2)*	1,20	0,376
1-(1-enyl-palmitoyl)-2-linoleoyl-GPC (P-16:0/18:2)*	1,20	0,507
isobutyrylcarnitine (C4)	1,20	0,610
1-linoleoyl-GPE (18:2)*	1,20	0,706
dihomo-linolenate (20:3n3 or n6)	1,20	0,834
cytosine	1,20	0,867
glycerophosphoserine*	1,19	0,260
S-(1,2-dicarboxyethyl)glutathione	1,19	0,283
UDP-glucose	1,19	0,289
N6,N6-dimethyllysine	1,19	0,430
1-(1-enyl-palmitoyl)-2-oleoyl-GPC	1,19	0,556

(P-16:0/18:1)*		
biopterin	1,19	0,686
3-hydroxypalmitoylcarnitine	1,19	0,794
methylsuccinate	1,18	0,246
adenine	1,18	0,382
sphingosine	1,18	0,437
1,2-dilinoleoyl-GPC (18:2/18:2)	1,18	0,506
ophthalmate	1,18	0,608
glycerophosphorylcholine (GPC)	1,18	0,684
creatinine	1,18	0,708
1-oleoyl-GPI (18:1)	1,18	0,899
N-acetyltaurine	1,17	0,422
1-palmitoleoyl-2-oleoyl-GPE (16:1/18:1)*	1,17	0,434
pyridoxamine	1,17	0,466
1,2-dipalmitoyl-GPE (16:0/16:0)*	1,17	0,635
pyridoxamine phosphate	1,17	0,760
argininosuccinate	1,16	0,313
glycosyl-N-nervonoyl-sphingosine (d18:1/24:1)*	1,16	0,347
inosine	1,16	0,351
sphingomyelin (d18:2/24:2)*	1,16	0,630
4-hydroxyglutamate	1,16	0,668
N-glycolylneuraminate	1,15	0,356
2R,3R-dihydroxybutyrate	1,15	0,474
guanosine	1,15	0,532
1-palmitoleoyl-2-linoleoyl-GPC (16:1/18:2)*	1,15	0,607
ceramide (d18:1/14:0, d16:1/16:0)*	1,15	0,772
alpha-tocopherol	1,15	0,998
sphingomyelin (d18:1/22:2, d18:2/22:1, d16:1/24:2)*	1,14	0,295
glycylvaline	1,14	0,356
3-hydroxy-3-methylglutarate	1,14	0,361
N6-succinyladenosine	1,14	0,516
dihydrobiopterin	1,14	0,699
1-stearoyl-2-oleoyl-GPE (18:0/18:1)	1,14	0,774
1-margaroylglycerol (17:0)	1,14	0,807
thymidine	1,13	0,385
2-dimethylaminoethanol	1,13	0,400
xanthosine 5'-monophosphate (xmp)	1,13	0,470
glycylisoleucine	1,13	0,482
1-palmitoyl-2-oleoyl-GPG (16:0/18:1)	1,13	0,594
1-(1-enyl-palmitoyl)-2-palmitoleoyl-	1,13	0,614

GPC (P-16:0/16:1)*		
flavin mononucleotide (FMN)	1,13	0,897
S-methylglutathione	1,12	0,469
1-oleoyl-2-linoleoyl-GPE (18:1/18:2)*	1,12	0,566
7-hydroxycholesterol (alpha or beta)	1,12	0,576
1-(1-enyl-palmitoyl)-2-palmitoyl-GPC (P-16:0/16:0)*	1,12	0,580
1-(1-enyl-palmitoyl)-2-arachidonoyl-GPE (P-16:0/20:4)*	1,12	0,651
glycosyl ceramide (d18:1/20:0, d16:1/22:0)*	1,12	0,710
1,2-dioleoyl-GPG (18:1/18:1)	1,12	0,809
(16 or 17)-methylstearate (a19:0 or i19:0)	1,12	0,976
uridine	1,11	0,303
glutarate (C5-DC)	1,11	0,503
glutamate	1,11	0,519
3-hydroxydecanoylcarnitine	1,11	0,525
caproate (6:0)	1,11	0,665
phosphoethanolamine	1,11	0,669
1-palmitoyl-2-gamma-linolenoyl-GPC (16:0/18:3n6)*	1,11	0,695
beta-guanidinopropanoate	1,11	0,833
1-pentadecanoylglycerol (15:0)	1,11	0,966
pipecolate	1,10	0,316
tryptophan	1,10	0,349
asparagine	1,10	0,400
N6-acetyllysine	1,10	0,549
prolylglycine	1,10	0,553
sphinganine	1,10	0,621
1-docosahexaenoylglycerol (22:6)	1,10	0,627
5-methylthioadenosine (MTA)	1,10	0,630
3-hydroxyhexanoylcarnitine (1)	1,10	0,651
1-(1-enyl-stearoyl)-2-arachidonoyl-GPE (P-18:0/20:4)*	1,10	0,824
ceramide (d16:1/24:1, d18:1/22:1)*	1,10	0,838
ceramide (d18:1/20:0, d16:1/22:0, d20:1/18:0)*	1,10	0,954
taurine	1,09	0,434
lactosyl-N-palmitoyl-sphingosine (d18:1/16:0)	1,09	0,492
guanidinoacetate	1,09	0,657
1-oleoyl-2-arachidonoyl-GPI (18:1/20:4)*	1,09	0,682
1-(1-enyl-stearoyl)-2-oleoyl-GPE (P-18:0/18:1)	1,09	0,783

N-palmitoyl-sphingosine (d18:1/16:0)	1,09	0,959
alanine	1,08	0,428
histidine	1,08	0,510
gulonate*	1,08	0,538
lactosyl-N-nervonoyl-sphingosine (d18:1/24:1)*	1,08	0,542
nicotinamide	1,08	0,586
malate	1,08	0,591
lactosyl-N-behenoyl-sphingosine (d18:1/22:0)*	1,08	0,643
1-palmitoyl-2-arachidonoyl-GPI (16:0/20:4)*	1,08	0,649
benzoate	1,08	0,673
tryptophylglycine	1,08	0,752
hexadecasphingosine (d16:1)*	1,08	0,791
1-stearoyl-2-arachidonoyl-GPC (18:0/20:4)	1,08	0,881
(14 or 15)-methylpalmitate (a17:0 or i17:0)	1,08	0,908
N-palmitoyl-sphingadienine (d18:2/16:0)*	1,08	0,916
1-(1-enyl-stearoyl)-GPE (P-18:0)*	1,08	0,934
1-arachidonoyl-GPI (20:4)*	1,08	0,943
1-palmitoyl-GPI (16:0)	1,08	0,978
taurochenodeoxycholate	1,08	0,979
serine	1,07	0,452
methionine	1,07	0,492
sphingomyelin (d18:2/23:1)*	1,07	0,655
tyrosylglycine	1,07	0,671
sphingomyelin (d17:1/16:0, d18:1/15:0, d16:1/17:0)*	1,07	0,682
sphingomyelin (d18:2/21:0, d16:2/23:0)*	1,07	0,691
trigonelline (N'-methylnicotinate)	1,07	0,696
gamma-glutamylglutamate	1,07	0,698
cytidine 5'-diphosphocholine	1,07	0,723
1-palmitoyl-2-oleoyl-GPE (16:0/18:1)	1,07	0,732
pantothenate	1,07	0,737
phytosphingosine	1,07	0,756
1-(1-enyl-palmitoyl)-2-oleoyl-GPE (P-16:0/18:1)*	1,07	0,758
1,2-dioleoyl-GPC (18:1/18:1)	1,07	0,772
1-stearoyl-2-docosahexaenoyl-GPC (18:0/22:6)	1,07	0,868
sphingomyelin (d18:1/25:0, d19:0/24:1, d20:1/23:0, d19:1/24:0)*	1,07	0,888

1-stearoyl-GPG (18:0)	1,07	0,943
tyrosine	1,06	0,565
5-(2-Hydroxyethyl)-4-methylthiazole	1,06	0,570
pyridoxate	1,06	0,572
carnitine	1,06	0,589
guanine	1,06	0,629
choline phosphate	1,06	0,639
1-palmitoyl-2-arachidonoyl-GPE (16:0/20:4)*	1,06	0,699
1,2-dioleoyl-GPE (18:1/18:1)	1,06	0,753
3'-dephospho-CoA-glutathione*	1,06	0,775
ergothioneine	1,06	0,801
behenoyl sphingomyelin (d18:1/22:0)*	1,06	0,824
lignoceroyl sphingomyelin (d18:1/24:0)	1,06	0,840
1-(1-enyl-palmitoyl)-2-arachidonoyl-GPC (P-16:0/20:4)*	1,06	0,891
glycochenodeoxycholate	1,06	0,945
lactose	1,06	0,984
threonine	1,05	0,527
proline	1,05	0,607
arginine	1,05	0,618
phenylalanine	1,05	0,620
lysine	1,05	0,635
spermidine	1,05	0,689
isoleucine	1,05	0,707
uridine 5'-monophosphate (UMP)	1,05	0,763
1-palmitoyl-2-palmitoleoyl-GPC (16:0/16:1)*	1,05	0,790
hypotaurine	1,05	0,806
pyridoxal	1,05	0,816
1-oleoyl-2-docosahexaenoyl-GPC (18:1/22:6)*	1,05	0,842
N('1)-acetylspermidine	1,05	0,862
succinate	1,05	0,934
1-stearoyl-2-arachidonoyl-GPE (18:0/20:4)	1,05	0,941
margaroylcarnitine (C17)*	1,05	0,958
1-myristoyl-2-arachidonoyl-GPC (14:0/20:4)*	1,05	0,983
1-(1-enyl-palmitoyl)-GPE (P-16:0)*	1,05	0,997
creatine	1,04	0,631
deoxycarnitine	1,04	0,667
glycerophosphoinositol*	1,04	0,669
gamma-glutamyl-epsilon-lysine	1,04	0,676
leucine	1,04	0,724

5-(galactosylhydroxy)-L-lysine	1,04	0,740
butyrate (4:0)	1,04	0,771
fructose	1,04	0,837
glycerophosphoglycerol	1,04	0,841
1-myristoyl-2-palmitoyl-GPC (14:0/16:0)	1,04	0,876
pantetheine	1,04	0,977
putrescine	1,04	0,985
creatine phosphate	1,04	0,989
inosine 5'-monophosphate (IMP)	1,03	0,656
myo-inositol	1,03	0,731
1-palmitoyl-2-oleoyl-GPS (16:0/18:1)	1,03	0,770
cysteinylglycine	1,03	0,784
1-(1-enyl-oleoyl)-2-oleoyl-GPE (P-18:1/18:1)*	1,03	0,787
N-acetylmethionine	1,03	0,800
phenylalanylglycine	1,03	0,832
3-hydroxyoleoylcarnitine	1,03	0,886
1-oleoyl-2-docosahexaenoyl-GPE (18:1/22:6)*	1,03	0,886
1-(1-enyl-oleoyl)-GPE (P-18:1)*	1,03	0,890
N-behenoyl-sphingadienine (d18:2/22:0)*	1,03	0,902
N-nervonoyl-sphingadiene (d18:2/24:1)*	1,03	0,904
thiamin (Vitamin B1)	1,03	0,906
glycerophosphoethanolamine	1,03	0,978
sphingomyelin (d18:2/18:1)*	1,02	0,714
glycerol	1,02	0,748
phenol glucuronide	1,02	0,768
phenol red	1,02	0,817
valine	1,02	0,818
2-hydroxyadipate	1,02	0,822
palmitoylcarnitine (C16)	1,02	0,830
palmitoyl dihydrosphingomyelin (d18:0/16:0)*	1,02	0,830
1,2-dipalmitoyl-GPC (16:0/16:0)	1,02	0,837
1-methylnicotinamide	1,02	0,885
ribonate	1,02	0,893
4-hydroxy-nonenal-glutathione	1,02	0,938
1-palmitoyl-2-stearoyl-GPC (16:0/18:0)	1,02	0,943
dimethylarginine (SDMA + ADMA)	1,02	0,951
methylmalonate (MMA)	1,02	0,963
alpha-ketoglutarate	1,02	0,963
1-stearoyl-2-oleoyl-GPS (18:0/18:1)	1,02	0,979

glycosyl-N-stearoyl-sphingosine (d18:1/18:0)	1,02	0,994
S-1-pyrroline-5-carboxylate	1,01	0,693
1-stearoyl-GPE (18:0)	1,01	0,708
1-palmitoleoyl-GPC (16:1)*	1,01	0,730
1-oleoyl-GPC (18:1)	1,01	0,761
saccharopine	1,01	0,762
cytidine 5'-monophosphate (5'-CMP)	1,01	0,814
phosphate	1,01	0,822
gamma-glutamylmethionine	1,01	0,827
N-acetylserine	1,01	0,874
tiglylcarnitine (C5:1-DC)	1,01	0,875
methionine sulfoxide	1,01	0,896
ribitol	1,01	0,939
1-methyl-5-imidazoleacetate	1,01	0,941
(R)-3-hydroxybutyrylcarnitine	1,01	0,942
stearoyl-docosahexaenoyl-glycerol (18:0/22:6) [2]*	1,01	0,948
1-palmitoyl-2-docosahexaenoyl-GPC (16:0/22:6)	1,01	0,950
S-methylcysteine	1,01	0,958
sphingomyelin (d18:1/14:0, d16:1/16:0)*	1,01	0,975
1-palmitoyl-2-arachidonoyl-GPC (16:0/20:4n6)	1,01	0,978
nicotinamide ribonucleotide (NMN)	1,01	0,983
1-oleoyl-2-arachidonoyl-GPE (18:1/20:4)*	1,01	0,993
1-oleoyl-GPE (18:1)	1,00	0,755
nicotinamide adenine dinucleotide (NAD+)	1,00	0,780
1-palmitoyl-GPE (16:0)	1,00	0,790
hypoxanthine	1,00	0,851
cytidine	1,00	0,869
S-methylmethionine	1,00	0,887
O-methyltyrosine	1,00	0,918
cholesterol	1,00	0,931
sphingomyelin (d18:1/20:0, d16:1/22:0)*	1,00	0,934
N,N,N-trimethyl-5-aminovalerate	1,00	0,942
3-methyl-2-oxovalerate	1,00	0,945
N-acetylalanine	1,00	0,950
sphingomyelin (d18:2/16:0, d18:1/16:1)*	1,00	0,982
sphingomyelin (d18:2/24:1, d18:1/24:2)*	1,00	0,986
1-carboxyethylleucine	1,00	0,988

1-stearoyl-2-arachidonoyl-GPS (18:0/20:4)	1,00	0,997
1-palmitoyl-2-oleoyl-GPC (16:0/18:1)	1,00	0,999
mannose-6-phosphate	1,00	1,000
ribulose/xylulose	1,00	1,000
1-palmitoyl-2-docosahexaenoyl-glycosyl-GPE (16:0/22:6)**	1,00	1,000
adenosine 2'-monophosphate (2'-AMP)	1,00	1,000
pinosylvin monomethylether	1,00	1,000
4-hydroxyphenylpyruvate	0,99	0,730
glycerol 3-phosphate	0,99	0,822
1-stearoyl-2-arachidonoyl-GPI (18:0/20:4)	0,99	0,868
beta-alanine	0,99	0,886
N-acetylneuraminate	0,99	0,896
1-carboxyethylphenylalanine	0,99	0,899
1-myristoylglycerol (14:0)	0,99	0,916
2'-deoxyuridine	0,99	0,917
N6-carbamoylthreonyladenosine	0,99	0,918
4-guanidinobutanoate	0,99	0,926
choline	0,99	0,928
1-palmitoyl-2-docosahexaenoyl-GPE (16:0/22:6)*	0,99	0,933
S-adenosylmethionine (SAM)	0,99	0,943
3-methylcytidine	0,99	0,943
cysteine sulfinic acid	0,99	0,944
gamma-glutamylserine	0,99	0,949
gamma-glutamylalanine	0,99	0,949
sphingomyelin (d18:1/20:1, d18:2/20:0)*	0,99	0,954
N1-methyladenosine	0,99	0,967
S-adenosylhomocysteine (SAH)	0,99	0,970
behenoylcarnitine (C22)*	0,99	0,976
erythronate*	0,99	0,996
CoA-glutathione*	0,98	0,509
1-lignoceroyl-GPC (24:0)	0,98	0,596
1-(1-enyl-palmitoyl)-GPC (P-16:0)*	0,98	0,631
cysteine-glutathione disulfide	0,98	0,691
sphingomyelin (d17:1/14:0, d16:1/15:0)*	0,98	0,736
guanosine 5'- monophosphate (5'-GMP)	0,98	0,759
6-oxopiperidine-2-carboxylate	0,98	0,764
2-aminoheptanoate	0,98	0,766
arachidonoylcarnitine (C20:4)	0,98	0,825

1-palmitoyl-2-linoleoyl-GPC (16:0/18:2)	0,98	0,866
myristoyl dihydrosphingomyelin (d18:0/14:0)*	0,98	0,902
gamma-glutamyltryptophan	0,98	0,912
2'-deoxycytidine	0,98	0,916
1,2-dioleoyl-GPS (18:1/18:1)	0,98	0,947
thioproline	0,98	0,968
orotidine	0,98	0,969
N-acetylleucine	0,98	0,998
flavin adenine dinucleotide (FAD)	0,97	0,677
1-arachidonylglycerol (20:4)	0,97	0,681
1-stearoyl-2-oleoyl-GPC (18:0/18:1)	0,97	0,756
phenyllactate (PLA)	0,97	0,794
tricosanoyl sphingomyelin (d18:1/23:0)*	0,97	0,838
N-formylmethionine	0,97	0,859
sphingomyelin (d18:2/23:0, d18:1/23:1, d17:1/24:1)*	0,97	0,864
1-carboxyethylisoleucine	0,97	0,890
glutamine	0,97	0,891
allantoin	0,97	0,893
beta-citrylglutamate	0,97	0,915
N6,N6,N6-trimethyllysine	0,97	0,934
imidazole propionate	0,97	0,943
C-glycosyltryptophan	0,97	0,945
1-carboxyethylvaline	0,97	0,949
palmitoyl sphingomyelin (d18:1/16:0)	0,97	0,949
hydroxy-N6,N6,N6-trimethyllysine*	0,97	0,953
sphingomyelin (d18:1/22:1, d18:2/22:0, d16:1/24:1)*	0,97	0,966
carnosine	0,97	0,992
N-stearoyl-sphingosine (d18:1/18:0)*	0,96	0,649
oleoylcarnitine (C18:1)	0,96	0,670
3-hydroxymyristate	0,96	0,681
3-methyl-2-oxobutyrate	0,96	0,704
N-stearoyl-sphingadienine (d18:2/18:0)*	0,96	0,716
sphingomyelin (d18:1/21:0, d17:1/22:0, d16:1/23:0)*	0,96	0,716
N-palmitoyl-sphinganine (d18:0/16:0)	0,96	0,733
sphingomyelin (d18:1/19:0, d19:1/18:0)*	0,96	0,759
gamma-glutamylthreonine	0,96	0,834
3-hydroxyoctanoate	0,96	0,837

2-methylmalonylcarnitine (C4-DC)	0,96	0,861
sphingomyelin (d18:1/24:1, d18:2/24:0)*	0,96	0,863
uracil	0,96	0,893
5-aminovalerate	0,96	0,947
N6-methyllysine	0,96	0,954
stearoyl sphingomyelin (d18:1/18:0)	0,96	0,954
1-methylhistidine	0,96	0,958
arabonate/xylonate	0,96	0,970
2,3-dihydroxy-5-methylthio-4-pentenoate (DMTPA)*	0,96	0,987
carboxyethyl-GABA	0,96	0,990
N-acetylmethionine sulfoxide	0,95	0,691
trimethylamine N-oxide	0,95	0,764
glycine	0,95	0,766
N-acetylcysteine	0,95	0,839
sulfate*	0,95	0,844
N-acetylasparagine	0,95	0,880
methionine sulfone	0,95	0,990
stearoyl ethanolamide	0,94	0,563
1-palmitoyl-GPC (16:0)	0,94	0,568
thiamin diphosphate	0,94	0,637
pyruvate	0,94	0,679
7-methylguanine	0,94	0,730
benzoylcarnitine*	0,94	0,739
pyridoxine (Vitamin B6)	0,94	0,779
trans-4-hydroxyproline	0,94	0,797
N-acetylthreonine	0,94	0,799
4-cholesten-3-one	0,94	0,818
gamma-glutamylglutamine	0,94	0,849
xanthine	0,94	0,852
ethylmalonate	0,94	0,871
O-sulfo-L-tyrosine	0,94	0,893
ceramide (d18:1/17:0, d17:1/18:0)*	0,93	0,590
2-aminoadipate	0,93	0,595
kynurenine	0,93	0,654
3-hydroxyhexanoate	0,93	0,671
p-cresol sulfate	0,93	0,688
uridine 5'-diphosphate (UDP)	0,93	0,700
3'-dephosphocoenzyme A	0,93	0,701
ornithine	0,93	0,751
sphingomyelin (d18:1/17:0, d17:1/18:0, d19:1/16:0)	0,93	0,761
1-dihomo-linolenylglycerol (20:3)	0,93	0,764

gamma-glutamylvaline	0,93	0,790
betaine	0,93	0,814
3-sulfo-L-alanine	0,93	0,835
N-acetyl-glucosamine 1-phosphate	0,93	0,846
adenosine 3',5'-cyclic monophosphate (cAMP)	0,93	0,892
urate	0,93	0,914
mannonate*	0,93	0,933
UDP-glucuronate	0,93	0,961
sphingomyelin (d18:2/14:0, d18:1/14:1)*	0,92	0,482
sphingomyelin (d17:2/16:0, d18:2/15:0)*	0,92	0,508
adenosine 5'-monophosphate (AMP)	0,92	0,543
N-acetyl-aspartyl-glutamate (NAAG)	0,92	0,613
thiamin monophosphate	0,92	0,630
gamma-glutamylleucine	0,92	0,655
gamma-glutamylisoleucine*	0,92	0,722
sphingomyelin (d18:1/18:1, d18:2/18:0)	0,92	0,764
gamma-glutamyltyrosine	0,92	0,771
argininate*	0,92	0,864
1-methyl-4-imidazoleacetate	0,92	0,895
coenzyme A	0,91	0,412
1-carboxyethyltyrosine	0,91	0,417
homocysteine	0,91	0,539
cysteine	0,91	0,542
ribulonate/xylulonate/lyxonate*	0,91	0,566
N-stearoyl-sphinganine (d18:0/18:0)*	0,91	0,591
methylphosphate	0,91	0,595
4-methylcatechol sulfate	0,91	0,616
cystathionine	0,91	0,621
2'-O-methylcytidine	0,91	0,644
gamma-glutamylhistidine	0,91	0,657
palmitoylcholine	0,91	0,669
phosphopantetheine	0,91	0,694
1-stearoyl-2-linoleoyl-GPC (18:0/18:2)*	0,91	0,713
catechol sulfate	0,91	0,774
N-acetylvaline	0,91	0,774
5-methylcytidine	0,91	0,787
pyroglutamine*	0,91	0,800
5-oxoproline	0,91	0,872
myristoylcarnitine (C14)	0,90	0,534

gamma-glutamylphenylalanine	0,90	0,572
palmitoloelycholine	0,90	0,599
glycosyl-N-stearoyl-sphinganine (d18:0/18:0)*	0,90	0,636
cytidine-5'-diphosphoethanolamine	0,90	0,650
phenol sulfate	0,90	0,658
stachydrine	0,90	0,681
glutarylcarnitine (C5-DC)	0,90	0,775
xanthosine	0,90	0,853
beta-hydroxyisovalerate	0,90	0,854
indolelactate	0,89	0,495
palmitoleoylcarnitine (C16:1)*	0,89	0,512
N-acetylisoleucine	0,89	0,678
N-acetylglutamine	0,89	0,724
5-hydroxylysine	0,89	0,795
isovalerate (i5:0)	0,89	0,816
alanylleucine	0,89	0,823
phenylacetylglycine	0,89	0,971
sphingomyelin (d18:0/20:0, d16:0/22:0)*	0,88	0,410
behenoyl dihydrosphingomyelin (d18:0/22:0)*	0,88	0,473
N-acetylaspartate (NAA)	0,88	0,508
2-hydroxyglutarate	0,88	0,597
3-(4-hydroxyphenyl)lactate	0,88	0,624
arachidoylcarnitine (C20)*	0,88	0,694
3-hydroxyisobutyrate	0,88	0,701
desmosterol	0,88	0,734
citrulline	0,88	0,756
adenylosuccinate	0,88	0,779
imidazole lactate	0,87	0,433
sphingomyelin (d18:0/18:0, d19:0/17:0)*	0,87	0,455
bilirubin (Z,Z)	0,87	0,472
1-palmitoyl-2-linoleoyl-GPE (16:0/18:2)	0,87	0,487
S-carboxymethyl-L-cysteine	0,87	0,586
gamma-glutamyl-alpha-lysine	0,87	0,588
alpha-hydroxyisovalerate	0,87	0,595
pseudouridine	0,87	0,624
3-amino-2-piperidone	0,87	0,650
cytidine 5'-monophospho-N-acetylneuraminic acid	0,87	0,681
cysteine s-sulfate	0,87	0,726
arachidonoyl CoA	0,86	0,431
gamma-glutamylcysteine	0,86	0,449

riboflavin (Vitamin B2)	0,86	0,454
pro-hydroxy-pro	0,86	0,475
N-acetylglutamate	0,86	0,563
N-acetylarginine	0,86	0,579
2-methylcitrate/homocitrate	0,86	0,586
eicosenoylcarnitine (C20:1)*	0,86	0,633
propionylcarnitine (C3)	0,86	0,641
N,N,N-trimethyl-alanylproline betaine (TMAP)	0,85	0,253
UDP-N-acetylglucosamine/galactosamine	0,85	0,402
3-hydroxydecanoate	0,85	0,438
glutamate, gamma-methyl ester	0,85	0,524
stearoylcarnitine (C18)	0,85	0,536
2-arachidonoylglycerol (20:4)	0,85	0,591
N-acetylphenylalanine	0,85	0,658
penicillin G	0,85	0,778
lactate	0,84	0,315
3-methylhistidine	0,84	0,382
N2-acetyllysine	0,84	0,484
erucoylcarnitine (C22:1)*	0,84	0,565
N-acetylhistidine	0,84	0,620
oleoyl-arachidonoyl-glycerol (18:1/20:4) [2]*	0,83	0,339
oleoylcholine	0,83	0,382
UDP-galactose	0,83	0,389
arachidonoylcholine	0,83	0,423
mannitol/sorbitol	0,83	0,423
homoarginine	0,83	0,456
histidylalanine	0,83	0,501
1-stearoyl-GPC (18:0)	0,83	0,502
3-hydroxybutyroylglycine**	0,83	0,586
acetylcarnitine (C2)	0,82	0,415
phenylalanylalanine	0,82	0,481
N-acetylglucosamine 6-phosphate	0,82	0,504
isoleucylhydroxyproline*	0,82	0,512
1-meadoylglycerol (20:3n9)*	0,82	0,555
adenosine	0,81	0,330
hippurate	0,81	0,358
valylleucine	0,81	0,404
N-delta-acetylornithine	0,81	0,462
gamma-glutamylglycine	0,81	0,482
5-methyltetrahydrofolate (5MeTHF)	0,81	0,498
2-oleoylglycerol (18:1)	0,81	0,500

N-acetyltyrosine	0,81	0,524
retinol (Vitamin A)	0,80	0,160
palmitoyl-arachidonoyl-glycerol (16:0/20:4) [2]*	0,80	0,277
2-hydroxybutyrate/2-hydroxyisobutyrate	0,80	0,354
cysteinylglycine disulfide*	0,80	0,364
S-(3-hydroxypropyl)mercapturic acid (HPMA)	0,80	0,499
1,2-dilinoleoyl-GPE (18:2/18:2)*	0,80	0,558
5,6-dihydrouridine	0,79	0,262
homostachydrine*	0,79	0,400
valylglutamine	0,79	0,553
linoleoylcholine*	0,78	0,376
3-methoxytyrosine	0,77	0,236
dimethylglycine	0,77	0,236
S-lactoylglutathione	0,77	0,328
pyridoxine phosphate	0,77	0,348
lanthionine	0,77	0,685
leucylglutamine*	0,76	0,334
leucylglycine	0,76	0,862
4-ethylphenylsulfate	0,75	0,383
lysylleucine	0,75	0,979
stearoyl-arachidonoyl-glycerol (18:0/20:4) [2]*	0,74	0,267
betaine aldehyde	0,74	0,403
3-hydroxylaurate	0,73	0,107
threonylphenylalanine	0,73	0,223
2-docosahexaenoylglycerol (22:6)*	0,73	0,316
3-hydroxybutyrate (BHBA)	0,72	0,135
threonate	0,72	0,204
N2-acetyl,N6-methyllysine	0,72	0,212
3-aminoisobutyrate	0,72	0,235
N-acetyltryptophan	0,72	0,710
quinolinate	0,71	0,152
erythritol	0,70	0,236
guaiacol sulfate	0,70	0,345
2-hydroxyhippurate (salicylurate)	0,68	0,173
N-methyl-GABA	0,67	0,028
gamma-glutamylcitrulline*	0,67	0,486
4-acetamidobutanoate	0,66	0,012
cystine	0,63	0,382
taurocholate	0,60	0,087
1-linoleoyl-GPC (18:2)	0,59	0,504
arabitol/xylitol	0,58	0,053

laurylcarnitine (C12)	0,58	0,253
3-hydroxyhippurate	0,55	0,059
1-oleoylglycerol (18:1)	0,50	0,037
leucylalanine	0,48	0,085
myristoleoylcarnitine (C14:1)*	0,17	0,176
